# Unraveling endophytic diversity in dioecious *Siraitia grosvenorii*: implications for mogroside production

**DOI:** 10.1007/s00253-024-13076-8

**Published:** 2024-03-01

**Authors:** Anish Tamang, Amanpreet Kaur, Deepali Thakur, Ankita Thakur, Babit Kumar Thakur, Shivani, Mohit Swarnkar, Probir K. Pal, Vipin Hallan, Shiv Shanker Pandey

**Affiliations:** 1 Biotechnology Division, CSIR-Institute of Himalayan Bioresource Technology (IHBT), Palampur, 176061 HP India; 2 Agrotechnology Division, CSIR-Institute of Himalayan Bioresource Technology (IHBT), Palampur, 176061 HP India; 3 Academy of Scientific and Innovative Research (AcSIR), Ghaziabad, 201002 India

**Keywords:** Endophyte, Plant-endophyte interactions, Monk fruit plant, Dioecious plant, Metagenomics, Mogroside biosynthesis

## Abstract

**Abstract:**

Host and tissue-specificity of endophytes are important attributes that limit the endophyte application on multiple crops. Therefore, understanding the endophytic composition of the targeted crop is essential, especially for the dioecious plants where the male and female plants are different. Here, efforts were made to understand the endophytic bacterial composition of the dioecious *Siraitia grosvenorii* plant using 16S rRNA amplicon sequencing. The present study revealed the association of distinct endophytic bacterial communities with different parts of male and female plants. Roots of male and female plants had a higher bacterial diversity than other parts of plants, and the roots of male plants had more bacterial diversity than the roots of female plants. Endophytes belonging to the phylum *Proteobacteria* were abundant in all parts of male and female plants except male stems and fruit pulp, where the *Firmicutes* were most abundant. Class *Gammaproteobacteria* predominated in both male and female plants, with the genus *Acinetobacter* as the most dominant and part of the core microbiome of the plant (present in all parts of both, male and female plants). The presence of distinct taxa specific to male and female plants was also identified. *Macrococcus*, *Facklamia*, and *Propionibacterium* were the distinct genera found only in fruit pulp, the edible part of *S. grosvenorii*. Predictive functional analysis revealed the abundance of enzymes of secondary metabolite (especially mogroside) biosynthesis in the associated endophytic community with predominance in roots. The present study revealed bacterial endophytic communities of male and female *S. grosvenorii* plants that can be further explored for monk fruit cultivation, mogroside production, and early-stage identification of male and female plants.

**Key points:**

• *Male and female Siraitia grosvenorii plants had distinct endophytic communities*

• *The diversity of endophytic communities was specific to different parts of plants*

• *S. grosvenorii-associated endophytes may be valuable for mogroside biosynthesis and monk fruit cultivation*

**Supplementary Information:**

The online version contains supplementary material available at 10.1007/s00253-024-13076-8.

## Introduction

In nature, plants interact with different microbial communities inside and outside their tissues as endophytes and epiphytes (on the plant surface). Endophytes are the plant-associated microbes residing inside the plant tissue (inter- or intra-cellularly) without causing any harm to the host plant. Endophytes have special importance due to their direct and long-lasting interaction (within the plant tissue) with the host plant, which supports them in escaping from harsh environmental conditions (Pandey et al. 2022). Various studies have established the role of endophytes in plant growth and development. Endophytes have abilities to promote plant growth, provide protection to plants from environmental stress, improve biosynthesis of in-planta secondary metabolites, and are the source of important therapeutic compounds such as antibiotics, bioactive molecules, and volatile organic compounds (Tiwari et al. 2023; Gupta et al. 2020; Pandey et al. 2022; Rodriguez and Redman 2008; Schulz and Boyle 2005). Several endophytes that can produce bioactive secondary metabolites similar to their host plants have been identified. For instance, in *Podophyllum peltatum*, a podophyllotoxin-producing endophyte *Phialocephala fortinii* was identified (Eyberger et al. 2006). Likewise, in *Apodytes dimidiata*, the endophyte *Fusarium solani* was found to produce camptothecin, a crucial precursor for the synthesis of clinically useful anticancer drugs such as topotecan and irinotecan (Puri et al. 2005; Shweta et al. 2010). Additionally, an endophyte *Aspergillus fumigatus*, isolated from *Juniperus communis*, was found to produce deoxypodophyllotoxin (Kusari et al. 2009a). The endophyte *Eupenicillium parvum*, associated with *Azadirachta indica*, was identified as a producer of azadirachtin, a major tetranortriterpenoid. Furthermore, in *Hypericum perforatum*, an unidentified endophytic fungus was discovered to produce the host metabolites, namely emodin and hypericin (Kusari et al. 2008, 2009b). The endophytic fungus of *Taxus* plants, *Paraconiothyrium* sp., was reported to produce taxol (Soliman et al. 2011). Recent reports have also identified new endophytes producing taxol (Subban and Kempken 2023). Similarly, the vincristine and vinblastine-producing endophyte *Fusarium oxysporum* was isolated from *Catharanthus roseus* (Kumar et al. 2013).

Primarily, endophytes cause a positive impact on host plant health by making nutrient acquisition easy through increased phosphorus solubilization, nitrogen fixation, siderophore production, modulation of phytohormones content, and their biosynthesis in host plants, and also by providing protection from biotic and abiotic stresses (Miotto-Vilanova et al. 2016; Santoyo et al. 2016; Chhabra and Dowling 2017; Issa et al. 2018; Afzal et al. 2019; Molina-Montenegro et al. 2020). Recently, different studies have demonstrated the role of endophytes in the primary and secondary metabolism of plants, like the identification of endophytes promoting plant growth, and enhancing in-planta vindoline, ajmalicine, and serpentine biosynthesis in *Catharanthus roseus* (Tiwari et al. 2013; Pandey et al. 2016b; Singh et al. 2020). The role of endophytes in enhancing the withanolide A (WLA) and withaferin A (WFA) biosynthesis in *Withania somnifera* has also been reported (Pandey et al. 2018; Kushwaha et al. 2019a, b, c). Moreover, the WFA, which is usually found in leaves, was detected in the roots of the endophyte-treated plants, thus suggesting the role of endophytes in modulating the site of WFA biosynthesis in *W. somnifera* plant (Pandey et al. 2018). It has also been demonstrated that the consortium of different endophytes showed a cumulative effect by complimenting the functional deficiency of one endophyte for a specific effect by another endophyte (having deficient ability or function) for upregulating multiple genes of a metabolic pathway and enhanced the yield of pharmaceutically important metabolites (Ray et al. 2019). Recently, the compatibility of *Trichoderma viride* with the fungal endophytes of *W. somnifera* was also studied, and it was observed that the co-inoculation of *T. viride* along with inherent endophytes of *W. somnifera* enhanced the plant growth and in-planta withanolide accumulation (Kushwaha et al. 2019c). An endophytic fungus (*Aspergillus terreus*) of *W. somnifera* has been used as a biotic elicitor for increasing the biosynthesis of WLA in the root cell suspension culture of *W. somnifera* (Kushwaha et al. 2019b). Therefore, the endophytic community associated with a particular host plant has special importance as a source of potential microbes that may be used to promote plant growth, protect plants from abiotic and biotic stresses, and increase biosynthesis of secondary metabolites of host plants, and as an in-vitro platform for the production of host-similar-secondary metabolites or other therapeutic compounds. Endophytes are found to be associated with almost all plant species and are present in different parts of plants, including roots, leaves, stems, flowers, fruits, and seeds. Host and plant-tissue specificity are the key attributes of endophytic microbial communities that have been reported in several studies (Pandey et al. 2016a, 2022; Dastogeer et al. 2018; Fang et al. 2019; Li et al. 2020). In opium poppy, the plant-tissue specificity was shown with leaf-associated endophytes, improving plant photosynthesis, and capsule-associated endophytes (capsule is the site for the secondary metabolite biosynthesis in opium poppy), enhancing biosynthesis of benzylisoquinoline alkaloids (Pandey et al. 2016a). Host specificity of associated fungal endophytes was also shown in *Nicotiana* plants (Dastogeer et al. 2018). Host-specific attributes of endophytes were also demonstrated in *Acer pseudoplatanus* and *Fraxinus excelsior* (Schlegel et al. 2018). Therefore, the host and tissue specificity of endophytes are important aspects that need to be studied for a particular crop to select the most appropriate microbes to improve crop-specific desired traits.

*Siraitia grosvenorii*, commonly known as monk fruit, is a traditional Chinese medicinal plant used worldwide as a natural food sweetener. It is also used for the treatment of sore throat, lung congestion, and constipation. It is cultivated for its fruit, and its extract is almost 250 times sweeter than sugar (Itkin et al. 2016). Its intense sweet taste is due to the presence of mogrosides, which belong to the triterpenoid saponin class of compounds. Mogrosides are found to be involved in activities such as scavenging of reactive oxygen species (ROS), anti-inflammatory, anticancerous, regulation of lipid metabolism of blood, and improvement of blood glucose metabolism. Among different mogrosides present in the monk fruit, mogroside III E (MG IIIE) has the most intense sweetness, and it is also found to regulate blood glucose levels efficiently; however, its relative abundance is low compared to other types of mogroside (Chiu et al. 2020). The endophytic community associated with *S. grosvenorii* plant has not been much explored. Recently, two endophytic strains, *Diaporthe angelica* LHG-F5 and *F. solani* LHG-L4 were identified which were able to produce mogroside V (Bin et al. 2020). A fungal (*Ganoderma lucidum*)-mediated biotransformation of fruit extract to enrich MG III E by converting mogroside V to MG III E has also been reported (Chiu et al. 2020). Therefore, endophytes associated with *S. grosvenorii* plants may have promising potential for the improvement of host plant health and the production of mogroside.

*S. grosvenorii* plants are dioecious, having different male and female plants, and both types of plants are phenotypically very similar. Male and female plants can be only identified using very distinct phenotypes of male and female flowers. In addition, female plants also bear fruits. Therefore, here, efforts were made to understand the endophytic bacterial communities associated with different parts of male (roots, stems, leaves, and flowers) and female (roots, stems, leaves, flowers, fruits (fruit pulp and seeds)) *S. grosvenorii* plants through metagenomics using 16S rRNA amplicon sequencing approach.

## Material and method

### Plant materials and growing conditions

Different parts of plants (roots, leaves, stems, flowers, and fruits) were collected from male and female *S. grosvenorii* plants cultivated on the farm at CSIR-IHBT, Palampur ((32° 06′ 05″ N; 76° 34′ 10″ E) at an altitude of 1393 amsl) since 2018. Both male and female plants were randomly cultivated together, and the distance between the two plants was 2 m × 1.5 m. The details of the sampling layout are provided in Figure S1. Voucher specimens (male plant voucher specimen no. PLP22325 and female plant voucher specimen no. PLP22326) were stored at CSIR-IHBT, Palampur. Plant samples were carefully extracted using sterile gloves and shovels for 16S rRNA amplicon sequencing. For male *S. grosvenorii* plants, samples of the roots, stems, leaves, and flowers and, for female *S. grosvenorii* plants, samples of the roots, stems, leaves, flowers, and mature fruits (seeds and fruit pulp; 60 days after pollination) were collected in sterile bags. The collected samples were immediately taken to the laboratory for further processing. A total of 9 male plants and 9 female plants were taken for harvesting their samples of respective plant parts (Figure S1). Samples (each plant-part of a plant) of 3 plants were pooled and considered as one replicate, and a total of 3 replicates were used for sequencing for each plant-part of both male and female plants.

### Sample preparation

Surface-sterilized plant samples were used for DNA extraction for metagenomic investigation. Different tissues from male and female *S. grosvenorii* plants were separated using a sterile knife, then rinsed in sterile distilled water, and allowed to drain for 10 to 15 min. Surface sterilization of plant tissues was carried out according to the methods outlined by Nascimento et al. (2019) and Pandey et al. (2016a, b) with appropriate modifications. Plant tissues were washed with running tap water and then surface sterilized using the 1% (v/v) sodium hypochlorite solution. In brief, first, the plant tissues were immersed separately in sodium hypochlorite solution for 2–10 min (depending on the tissue type; leaf, stem, and flower tissues for 2–3 min and rhizome or root tissues for 5–8 min), followed by immersion in 70% ethanol (v/v) for 1 min. The disinfected tissues were thoroughly rinsed five times with sterile distilled water to remove the traces of ethanol from the tissue. All the sterilization procedures were done in aseptic conditions in the laminar flow hood. To validate the effectiveness of the surface sterilization procedure, the distilled water used in the final wash was inoculated on a nutrient agar plate and incubated at 28 °C in an incubator for 24 h. Bacterial growth (within 24 h of incubation) in the agar plates indicates inadequate surface sterilization; samples were discarded, and surface sterilization was repeated. After surface sterilization, tissues were stored at − 80 °C for further processing.

### DNA extraction and library preparation

Extraction of quality plant DNA is crucial for metagenome studies, enabling the investigation of the microbes inhabiting plants (Fadiji and Babalola 2020). Surface-sterilized plant tissues were ground separately with liquid nitrogen, and the total genomic DNA was extracted from the plant tissue samples using a Fast DNA™ SPIN Kit for Soil (MP Biomedicals, Santa Ana, CA, USA) following the manufacturer’s instructions. The quality (concentration and purity) of the extracted genomic DNA was tested at A_260_/A_280_ nm using Nanodrop (Thermo Fisher Scientific, Waltham, MA, USA) and Qubit™ 4.0 Fluorometer (Thermo Scientific, Waltham, MA, USA) and stored at − 20 °C for further processing.

The PCR library preparation was carried out using QIAseq 16S/ITS Region Panels PCR reaction kit (Qiagen, Hilden, Germany) and QIAseq 16S/ITS Region Panel Sample Index PCR Reaction kit (Qiagen, Hilden, Germany). The microbial genomic DNA extracted from plant tissues was normalized to a concentration of 1 ng/µl for amplification. PCR amplification was carried out using universal primers (forward primer: 5′-GCCTACGGGNGGCWGCAG-3′ and reverse primer: 5′-ACTACHVGGGTATCTAATCC-3′) that target V3-V4 hypervariable regions of the 16S rRNA gene using QIAseq 16S/ITS Panel Handbook in compliance with the manufacturer’s instructions.

PCR reactions were carried out with the initial denaturation at 95 °C for 2 min followed by 12 cycles of 95 °C for 30 s, 50 °C for 30 s and 72 °C for 2 min, and ended with a final extension step at 72 °C for 7 min. PCR amplicons were purified with QIAseq beads and further quantified with a Qubit™ 4.0 Fluorometer (Thermo Scientific, Waltham, MA, USA). The PCR amplicons were tagged with adapters for creating indexed libraries following the QIAseq 16S/ITS Panel Handbook (Qiagen, Hilden, Germany) according to the manufacturer’s instructions. Index PCR reactions were carried out with the initial denaturation at 95 °C for 2 min followed by 19 cycles of 95 °C for 30 s, 60 °C for 30 s, and 72 °C for 2 min and ended with a final extension at 72 °C for 7 min. Negative controls were included by replacing the template DNA with sterile MilliQ water. The quality of DNA libraries was checked using the Agilent 2100 Bioanalyzer system (Agilent, Santa Clara, CA, USA) and quantified by QIAGEN’s Library Quant System (Qiagen, Hilden, Germany). No amplicons were observed in negative controls; hence, they were not subjected to sequencing. The libraries were normalized and pooled before sequencing and then outsourced to T-CAG LifeSciences (New Delhi, India) for sequencing on the Illumina MiSeq (Illumina, San Diego, CA, USA) platform.

### Data analysis of 16S rRNA amplicon sequence

Demultiplexing of raw reads obtained from Illumina sequencing was done using the barcodes, and following that, the FastQC v0.11.9 (https://www.bioinformatics.babraham.ac.uk/projects /fastqc/) was performed to check the quality of the reads. Reads with a Phred score of less than 30 were trimmed using Cutadapt v3.4 (Martin 2011). The trimmed reads were then imported into the QIIME2 v2022.2 pipeline (Bolyen et al. 2019) to investigate the microbial composition and diversity. Before processing the samples for diversity analysis, the plant-associated taxa were removed manually from the OTU.biom file. The reads were denoised, and chimeric sequences were removed using DADA2 to give non-chimeric denoised paired-end reads (Callahan et al. 2016). Taxonomical assignment of reads was done based on the Greengenes v13_8 database using q2-feature-classifier (McDonald et al. 2012).

### Diversity index data analysis of amplicon

Alpha diversity indexes, including Chao1, Simpson, and Shannon, as well as observed species were evaluated in the R environment by implementing the phyloseq (McMurdie and Holmes 2013) and ggplot2 (v3.3.0) R packages (Wickham and Wickham 2016), whereas the beta diversity was determined by the Microbiome Analyst R server (Chong et al. 2020). OTUs (operational taxonomic units) that had a relative abundance of 1% or more were considered abundant. The QIIME-2 generated OTU table and taxonomic labels were uploaded to the Microbiome Analyst R server, where low count features (< 10% prevalence) were removed (to eliminate the sequencing error or low-level contamination), and rarefied to minimum library size for microbial diversity analysis. Analysis of variance (ANOVA) was used to determine significant differences in alpha diversity measures between different anatomical parts of male and female plants. Beta diversity was depicted using PCoA (principal coordinate analysis) plots, which were utilized to interpret differences in bacterial community composition across different parts of male and female plants, and NMDS (nonmetric multidimensional scaling) analysis was used to determine differences among or within the groups (Looft et al. 2012). Statistical difference was computed using the Bray–Curtis index with PERMANOVA and ANOSIM to test the significant compositional differences based on the categorical variable.

The linear discriminant analysis effect size (LEfSe) program was utilized to understand the differentially abundant taxa in different anatomical parts of male and female plants at an LDA (linear discriminant analysis) cut-off score ≥ 2 (Segata et al. 2011a). The significant groups of taxa were computed using the Kruskal–Wallis index, and then, LDA was applied to the taxa that met the significant threshold to deduce the effect size. The core microbiome was computed to identify the endophytic bacteria shared by the different anatomical parts of both male and female plants. There are two parameters to be considered while performing a core microbiome; the first one is sample prevalence, which is defined as the minimum fraction (percentage) of the sample in the community where the taxa must be observed. The other factor is the relative abundance (fraction) of the taxa, which determines the taxa to be taken as a part of the core member. The analysis and plots were generated using the Microbiome Analyst R server (Chong et al. 2020). Identification of important taxa and their correlations/co-occurrence patterns in different tissues of both male and female plants were analyzed using the univariate statistical methods (Segata et al. 2011b; Friedman and Alm 2012), which determined the significantly abundant microbial taxa in different parts of the plants.

### Functional prediction of metagenome data

The PICRUSt2 (Phylogenetic Investigation of Communities by Reconstruction of Unobserved States) was utilized to predict the potential function of the bacterial community found in different parts of both male and female plants (Douglas et al. 2020). PICRUSt2 predicts KEGG orthology (KOs) and Enzyme Commission numbers (EC numbers) and also their abundance based on the OTU matrix and 16sRNA marker sequences by utilizing the Kyoto Encyclopedia of Genes and Genomes (KEGG) database. KOs represent functional orthologous groups and facilitate the interpretation of biological pathways in the microbial community. Further, the genes involved in secondary metabolite biosynthesis were manually categorized based on the EC numbers.

## Results

### Distinct endophytic bacterial communities associated with male and female plants of *S. grosvenorii*

**Fig. 1:**
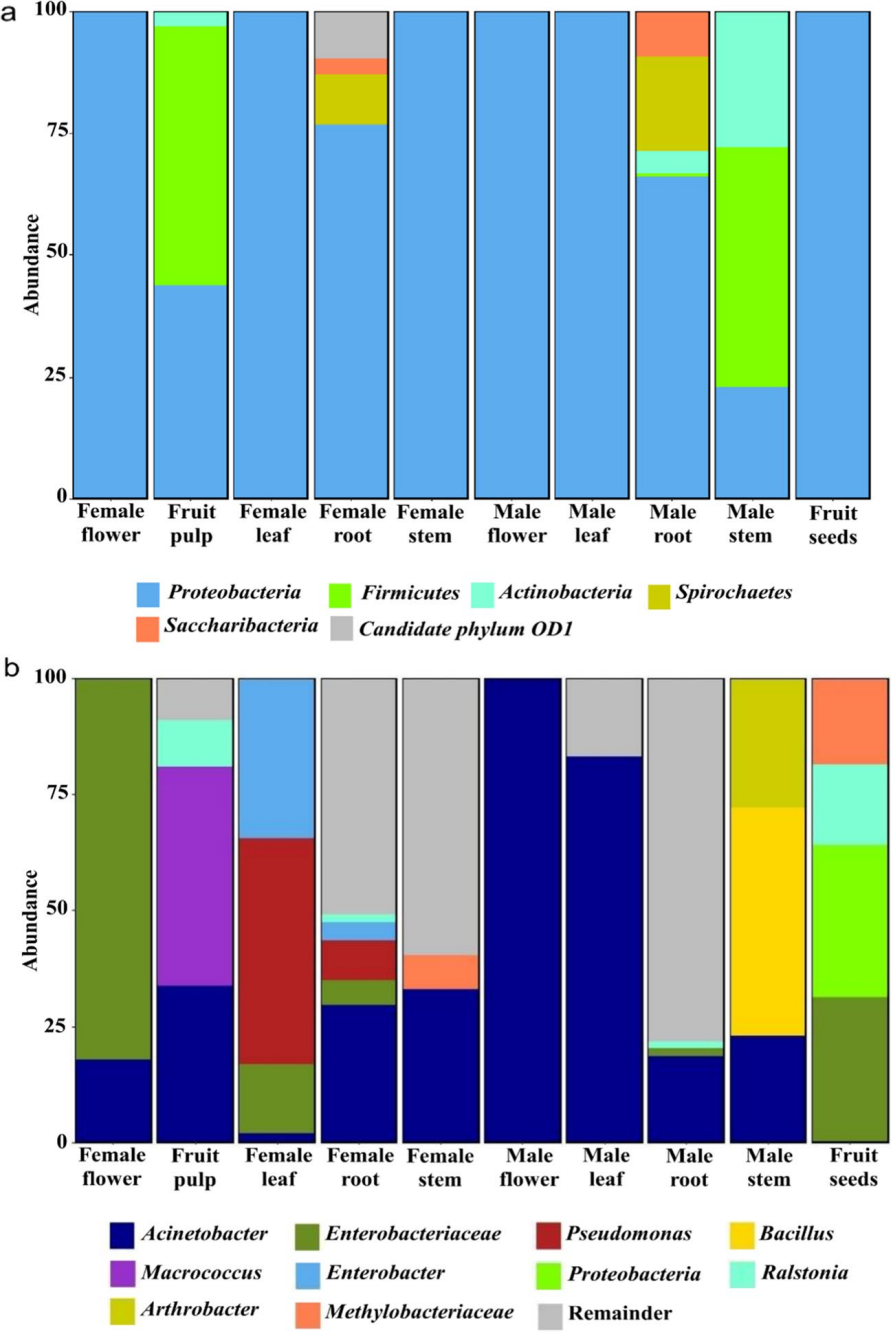
Relative abundance of different taxa at **a** phylum level and **b** genus level in different parts of male and female *S. grosvenorii* plants (taxa not identified at genus level are depicted at their higher taxa level)

It was observed that the roots of both male and female plants had the highest endophytic bacterial diversity compared to other parts of plants, and the roots of male plants had more bacterial endophytic diversity than the roots of female plants (Fig. 1, Table S1). Among the bacterial taxa diversity in the root tissues, *Herbaspirillum*, *Massilia*, *Lechevalieria*, *Mesorhizobium*, *Rhodomicrobium*, *Bacillus*, and *Pseudarthrobacter* were more predominant in male plants, whereas in the female plants, the abundant genera were *Pseudomonas*, *Enterobacter*, and *Escherichia-Shigella*. Leaves and flowers of male plants; stems, leaves, and flowers of female plants; and seeds of fruits were found to be associated with only endophytic bacteria belonging to phylum *Proteobacteria*. Moreover, roots of male and female plants, stems of male plants, and pulp of fruits were found to be associated with other phyla along with *Proteobacteria* (Fig. 1). Phylum *Proteobacteria* was abundant in all parts of male and female plants except male stems (23.02%) and pulp of fruits (43.8%), where the bacteria belonging to phylum *Firmicutes* (in pulp (53.03%) and male stem (49.21%)) were the most abundant.

Flowers of male plants and flowers and leaves of female plants harbored *Gammaproteobacteria*, while *Proteobacteria* associated with seeds include *Gammaproteobacteria*, *Betaproteobacteria*, and *Alphaproteobacteria* (Figure S2). Similarly, the bacteria belonging to phylum *Actinobacteria* were only present in roots (4.7%) and stems (27.78%) of male plants and pulp of fruits (3.17%) of female plants (Fig. 1). The phylum *Spirochetes* and *Saccharibacteria* (TM7) were found to be restricted to only roots of male and female plants (Fig. 1). *Parcubacteria* (Candidate phylum *OD1*) was present in female roots only. Interestingly, the genus *Acinetobacter* was found to be associated with all parts of male and female plants (Table S1). The male flowers had only endophytic bacteria belonging to the genus *Acinetobacter*, while the female flowers had *Acinetobacter* (17.74%) and *Enterobacteriaceae* (82.26%). In the leaves of male plants, *Acinetobacter* (83.18%) was most abundant, while *Pseudomonas* was most abundant in the leaves of female plants (48.65%). Endophytic bacteria belonging to *Pseudomonas*, *Enterobacter*, *Pantoea*, *OD1*, *Methylobacteriaceae*, *Methylotenera*, *Rheinheimera*, *Aeromonas*, *Macrococcus*, *Facklamia*, and *Propionibacterium* were only present in female plants (Table S1). Moreover, endophytic bacteria belonging to *Herbaspirillum*, *Duganella*, *Hyphomicrobiaceae*, *Sphingomonadaceae*, *Lentzea*, *Mesorhizobium*, *Rhodospirillaceae*, *Virgisporangium*, *Paracoccus*, *Legionellaceae*, *Turicibacter*, *Rhizobiales*, *Vogesella*, *Bacillus*, and *Arthrobacter* were found to be associated with male plants only (Table S1, Figure S3).

In addition, endophytes belonging to *Macrococcus*, *Facklamia*, *Propionibacterium*, and *Proteobacteria* (distinct from *Proteobacteria* present in other tissues) were only associated with fruit part, and *Macrococcus*, *Facklamia*, and *Propionibacterium* were the distinct genera present in fruit pulp which is the edible part of monk fruit plant. Besides, the leaves of male plants harbored bacterial endophytes belonging to the genus *Vogesella*, and the leaves of female plants had *Pseudomonas* and *Enterobacter*; therefore, these are the distinctive groups and may be used for early identification of male and female plants. This information revealed that the bacterial endophytic communities are distinct in different parts of female and male plants.

### Diversity indices among different parts of male and female plants

**Fig. 2:**
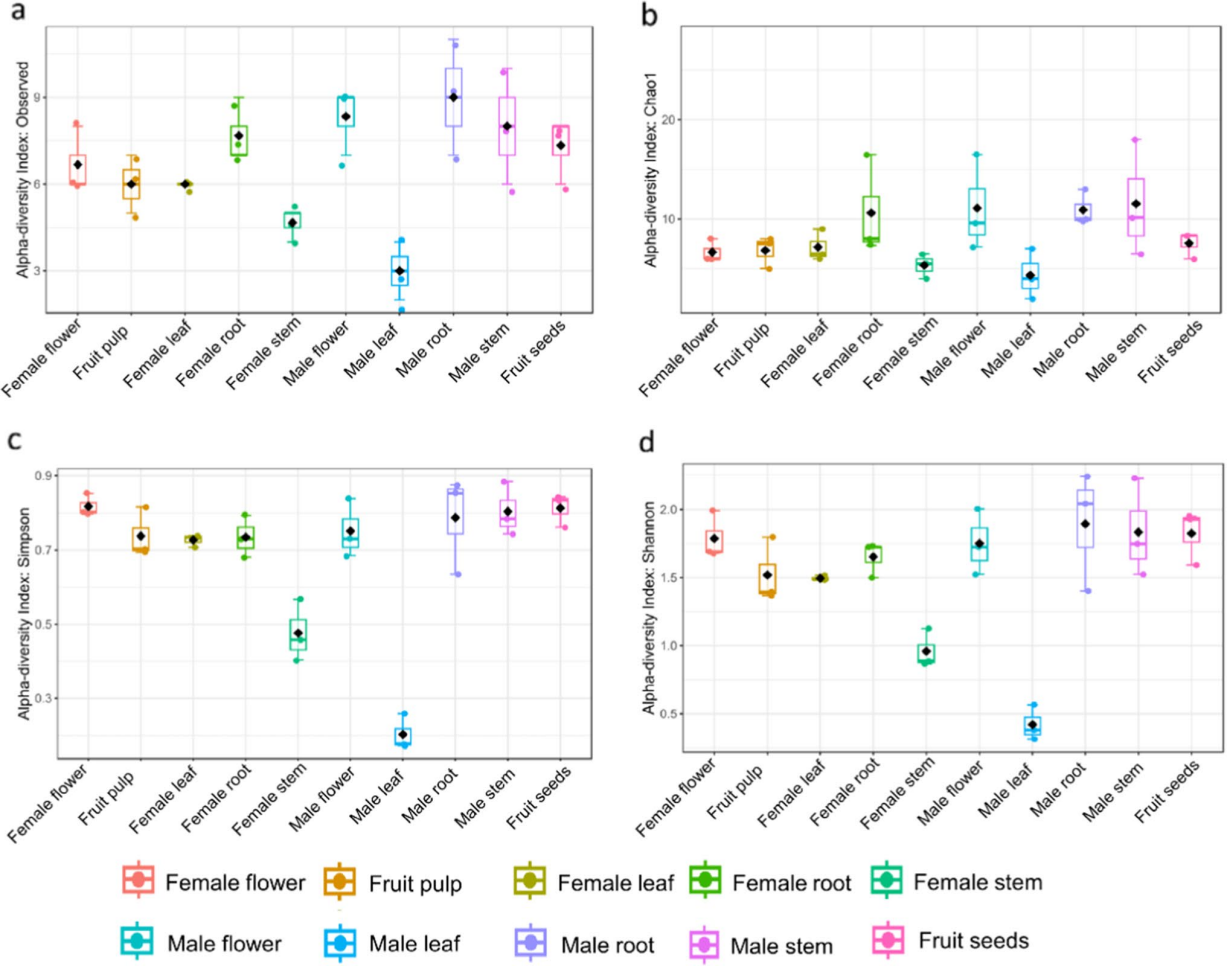
Alpha diversity based on **a** observed number of species, **b** Chao1, **c** Simpson, and **d** Shannon indexes. The *Y*-axes indicate the values for the corresponding index, and the *X*-axes indicate the different parts of male and female *S. grosvenorii* plants

In the case of roots, stems, leaves, and flowers of male and female plants, the alpha diversity was highest in roots, followed by stems, leaves, and flowers for various metrics, including the observed number of OTUs, Chao1, Simpson, and Shannon (Fig. 2). However, no significant difference was observed in alpha diversity between pulp and seeds of fruit samples. No significant difference was found when the alpha diversity of different parts of male plants was compared to the respective parts of female plants. Higher alpha diversity matrices of root tissues of male and female plants indicated more richness, diversity, and abundance of bacterial endophytes compared to other parts of plants. The statistical analysis was done with ANOVA to determine the significance level for Observed (*p* < 0.001, *F* = 6.34), Shannon (*p* < 0.001, *F* = 23.351), Simpson (*p* < 0.001, *F* = 23.35), and Chao1 (*p* = 0.09, *F* = 1.98). Whereas in the case of non-parametric test, the significance level was determined for Observed (*p* = 0.01, Kruskal–Wallis statistic = 21.43), Shannon (*p* = 0.02, Kruskal–Wallis statistic = 19.08), Simpson (*p* = 0.02, Kruskal–Wallis statistic = 18.86), and Chao1 (*p* = 0.05, Kruskal–Wallis statistic = 16.65) using Mann–Whitney test.

**Fig. 3:**
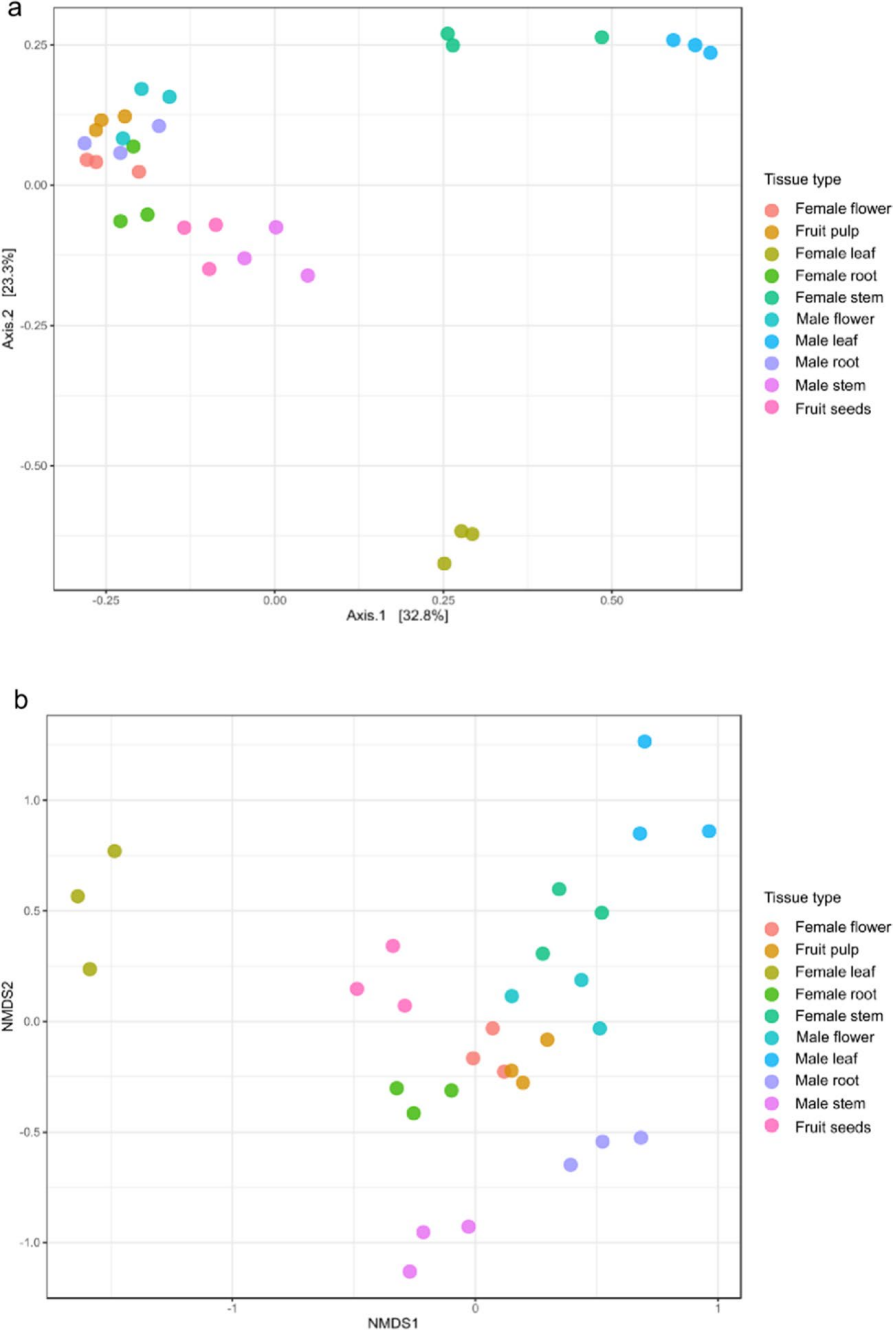
Beta diversity analysis. Two-dimensional scatter plots were generated using **a** PCoA based on unweighted UniFrac distance metric Bray Curtis. **b** NMDS. Samples corresponding to different parts of male and female *S. grosvenorii* plants were plotted as colored dots

Beta diversity measure plotted using PCoA revealed that the leaves of male plants and leaves and stems of female plants harbored phylogenetically distinct communities (Fig. 3a). Whereas female flowers, female fruits, female roots, male flowers, and male roots showed closed clustering. A nonmetric multidimensional scaling (NMDS) plot showed that microbial communities associated with female fruits and flowers showed close similarity, and communities of female roots, male flowers, and female stems formed distinct but close clusters, whereas communities in female leaves, male leaves, male stems, male roots, and seeds formed well-defined diverse clusters indicating different endophytic microbes residing in these tissues (Fig. 3b). Statistical analysis for PCoA and NMDS was done using Bray–Curtis index and estimated using PERMANOVA (permutational multivariate analysis of variance) which utilizes the distance (dissimilarity) between the samples of the same group and compares it with the distance between groups to estimate the significant difference between clusters of two groups. ANOSIM (analysis of group similarities) utilizes the ranks of all pairwise sample distances to determine whether with-in-group distances are greater or equal to between-group distances. The statistical analysis was done with PERMANOVA (*p* = 0.001, *R*^2^ = 0.83, *F* = 1.2) and ANOSIM (*p* < 0.001, *R*^2^ = 0.85).

### Determination of shared, distinct, and core taxa associated in different parts of male and female *S. grosvenorii* plants

**Fig. 4:**
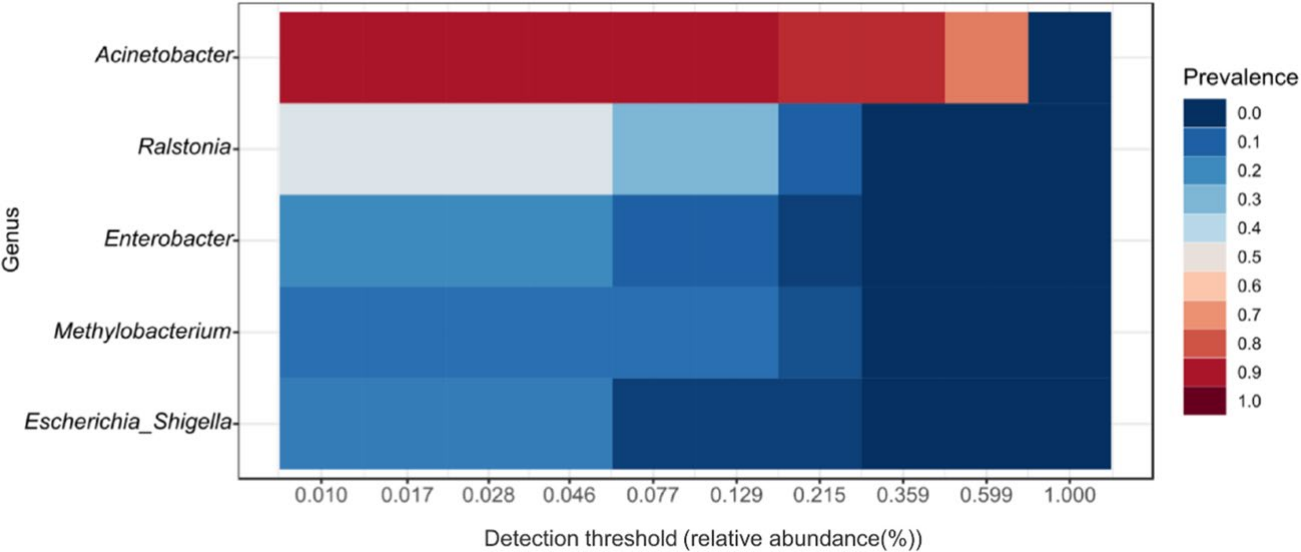
Core microbiota depicted as a heat map showing the absolute abundance of taxa for endophytes of *S. grosvenorii*

**Fig. 5:**
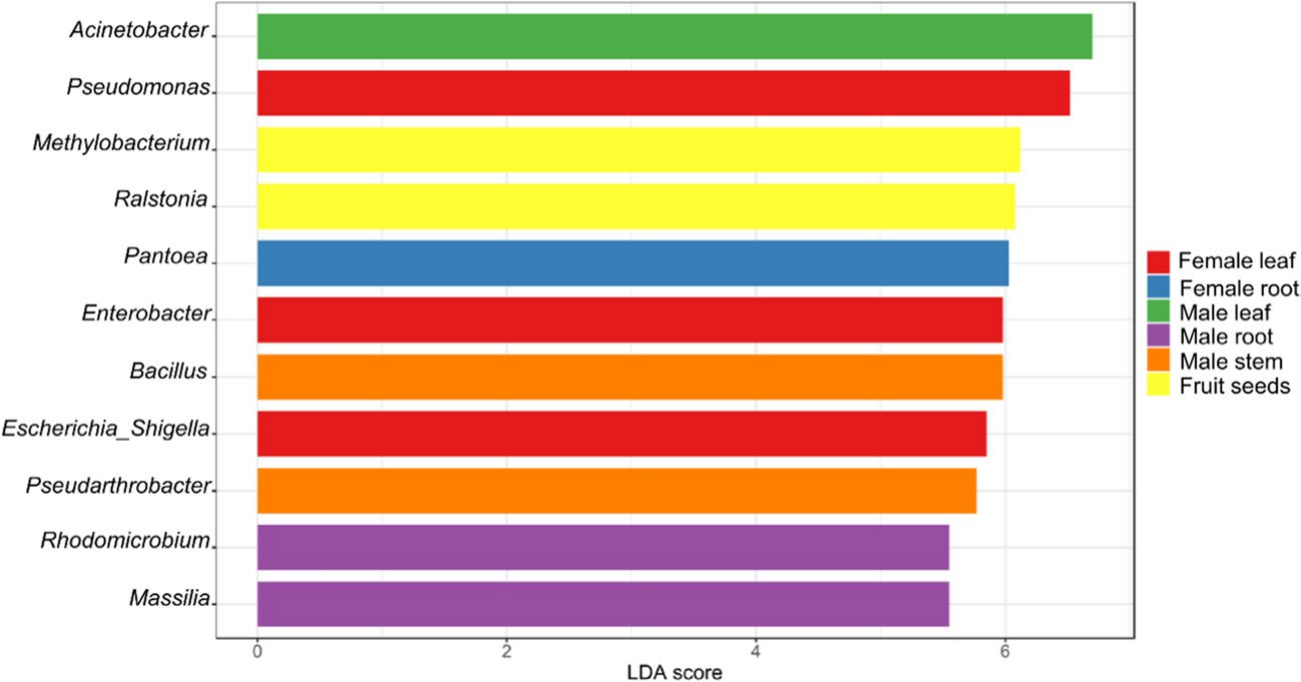
Differentially abundant bacterial taxa found in *S. grosvenorii*. Linear discriminant analysis (LDA) effect size (LEfSe) comparison of relative abundance. Horizontal bars represent the effect size for each taxon

**Fig. 6:**
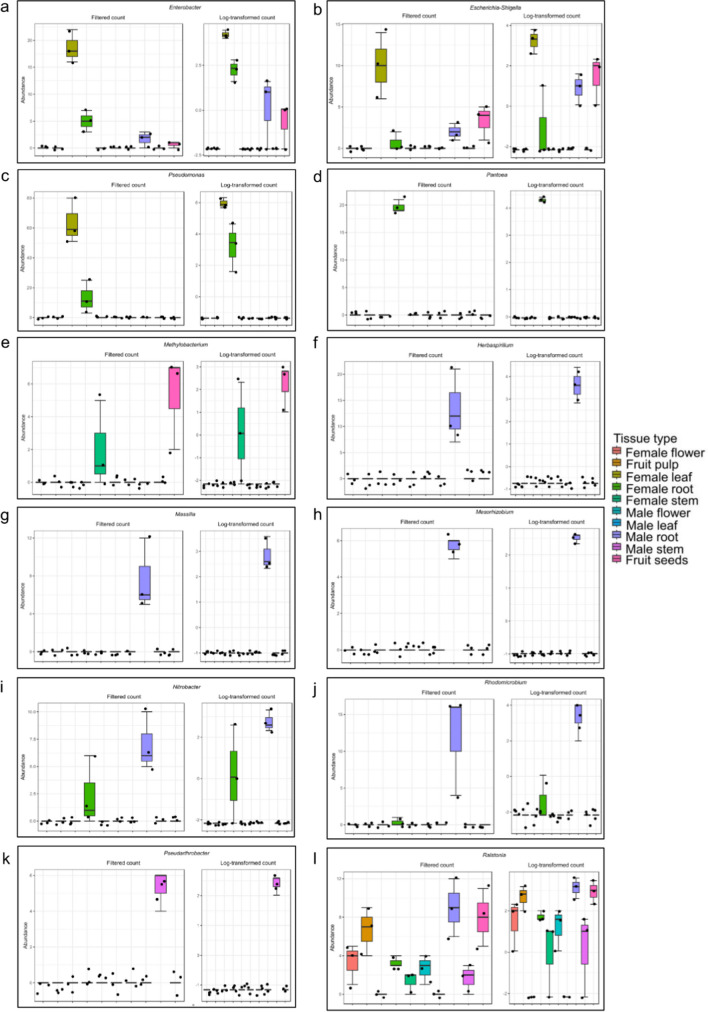
Univariate test analysis depicting significantly abundant taxa predominant in different parts of male and female *S. grosvenorii* plants

The core microbiome was computed to understand the taxa shared by the plants irrespective of the different tissues or sex. It was found that *Acinetobacter* was the most prevalent genus, followed by *Ralstonia*, *Enterobacter*, *Methylobacterium*, and *Escherichia-Shigella* (Fig. 4). Further, LEfSe analysis was performed to identify taxa that could be potential biomarkers for respective parts of male and female *S. grosvenorii* plants. It was observed that, in the male plants, *Acinetobacter* was predominant in the leaves, and *Bacillus* and *Pseudarthrobacter* in the stems, whereas for the roots, the predominant taxa were *Rhodomicrobium* and *Massilia* (Fig. 5). In the female plants, the predominant taxa in leaves were *Pseudomonas*, *Enterobacter*, and *Escherichia-Shigella*; *Pantoea* in the roots and seeds had *Methylobacterium* and *Ralstonia* as predominant taxa (Fig. 5; Table S2). The univariate analysis identified the significantly abundant taxa in different parts of male and female *S. grosvenorii* plants. Female leaves and root tissues showed a higher abundance of *Enterobacter* than their male counterparts (Fig. 6). Female leaf tissue exhibited abundance in *Escherichia-Shigella* and *Pseudomonas*. *Pantoea* was prominent in the female roots and stems, while *Methylobacterium* dominated in the seeds and female stems. In male roots, in addition to *Enterobacter* and *Escherichia-Shigella*, the *Herbaspirillum*, *Massilia*, *Mesorhizobium*, *Nitrobacter*, and *Rhodomicrobium* were abundant. *Pseudarthrobacter* was most prevalent in the male stems. The *Ralstonia* genus showed a higher abundance in the male roots and seeds compared to other parts of male and female *S. grosvenorii* plants (Fig. 6).

### Functional predictive analysis

**Fig. 7:**
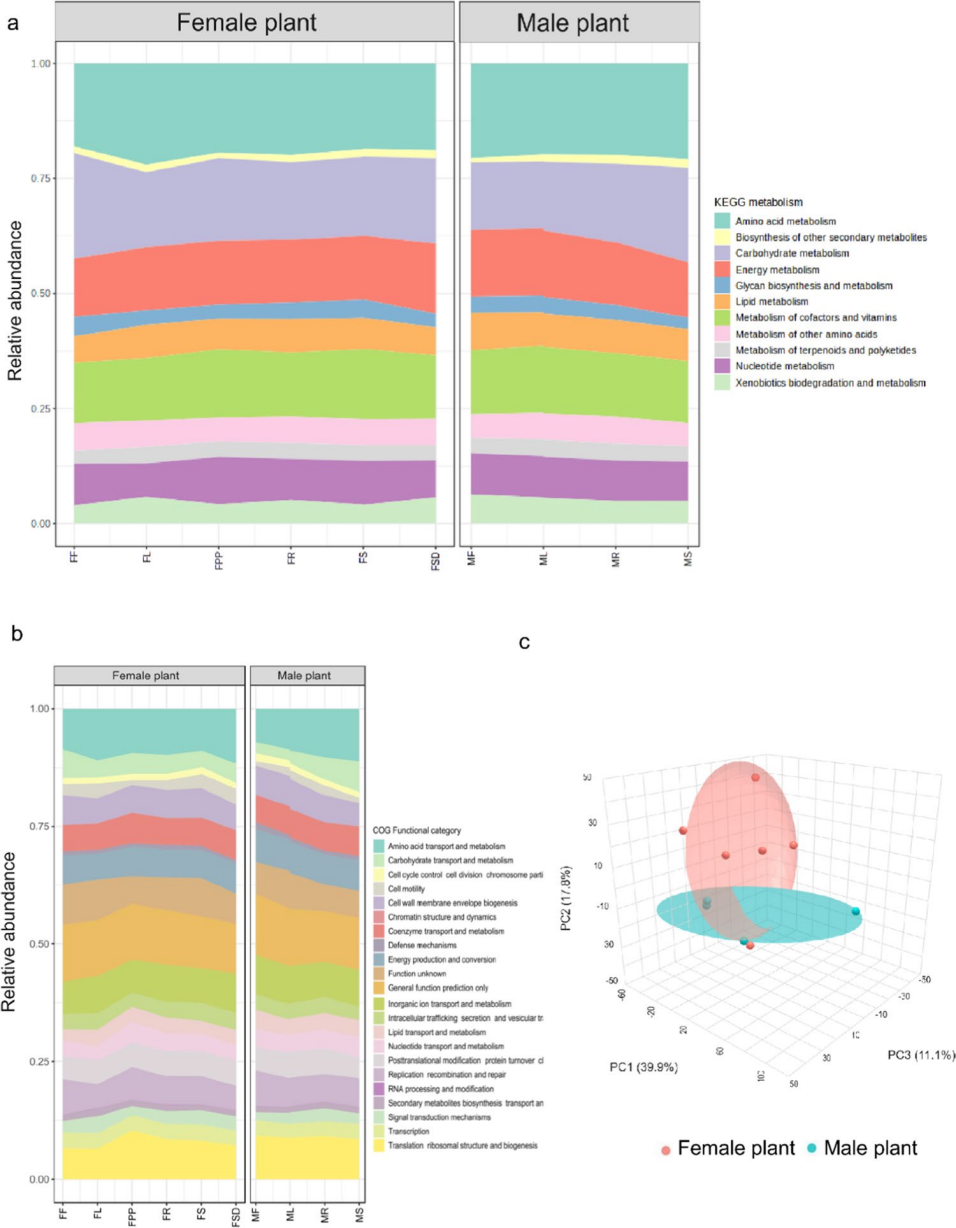
**a** KEGG (metabolism) pathway abundance, **b** COG functional category abundance in different parts of female and male plants, and **c** Principal component analysis (PCA) were used to depict the PICRUSt-predicted functional genes. FF, female flower; FL, female leaf; FPP, fruit pulp; FR, female root; FS, female stem; FSD, fruit seeds; MF, male flower; ML, male leaf; MR, male root; MS, male stem

**Fig. 8:**
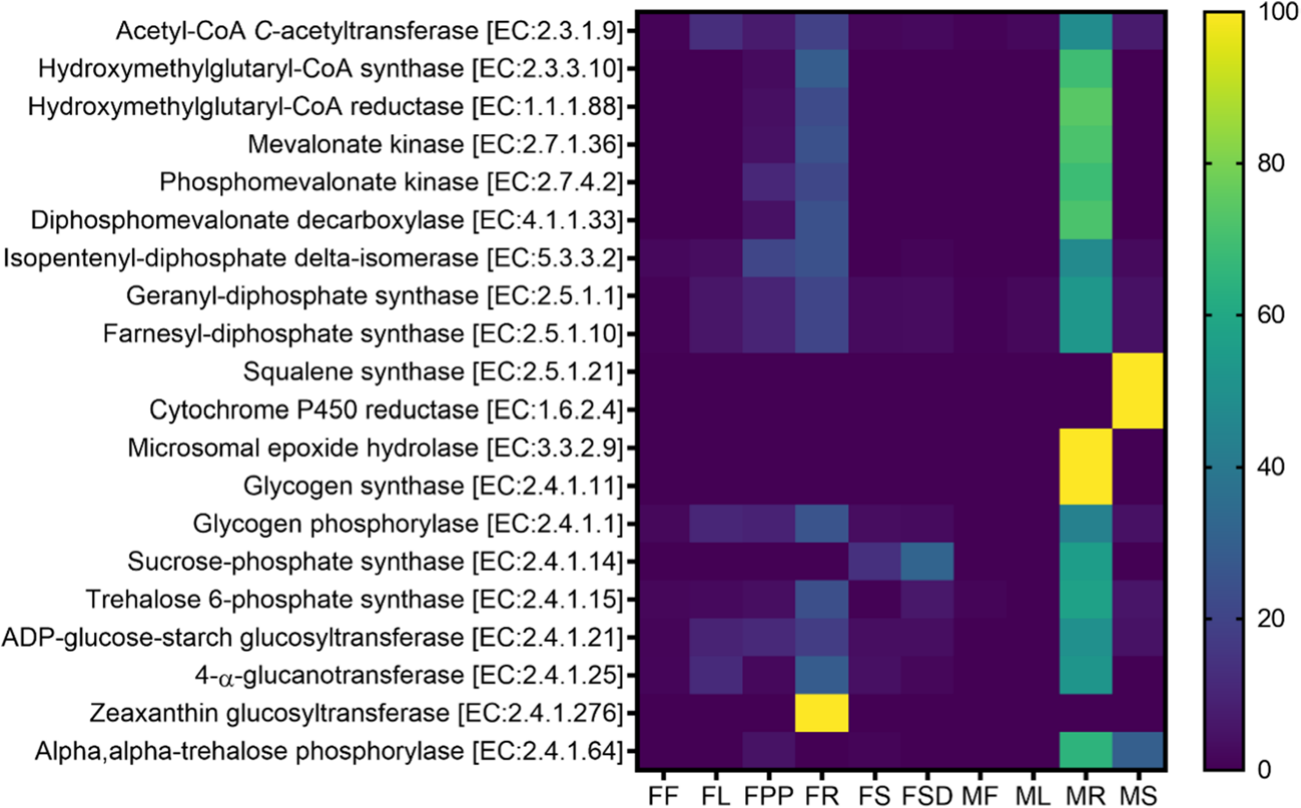
Heat map representation of secondary metabolite biosynthetic gene enrichment in different anatomical parts of female and male plants of *S. grosvenorii*. Genes are indicated in the diagram by encoded functions. FF, female flower; FL, female leaf; FPP, fruit pulp; FR, female root; FS, female stem; FSD, fruit seeds; MF, male flower; ML, male leaf; MR, male root; MS: male stem

The functional potential of the endophytic microbiota associated with different parts of male and female *S. grosvenorii* plants was predicted using PICRUSt (Phylogenetic Investigation of Communities by Reconstruction of Unobserved States) software. The KEGG metabolism (Fig. 7a) and COGs (Cluster of Orthologous Genes) (Fig. 7b) functional category were used to categorize the functional genes present in the endomicrobial communities of different parts of male and female *S. grosvenorii* plants. The predicted metabolism and functional categories included various pathways involved in the cellular and biological processes. Further, the genes responsible for secondary metabolite biosynthesis and metabolism of terpenoids and polyketides were also enriched in the microbial communities. KEGG pathway prediction revealed enrichment of various secondary metabolites pathways such as terpenoid backbone pathway, phenylpropanoid pathway, and carotenoid biosynthesis. (Table S3). Additional principal component analysis (PCA) conducted on the enriched functional genes present in both male and female plants also indicated the formation of distinct clusters among the enriched genes, highlighting the diversity of genes in male and female plants (Fig. 7c). The genes encoding enzymes involved in secondary metabolite biosynthesis were manually sorted based on the predicted genes. As mogrosides are triterpenoids, the enzymes involved in terpene backbone synthesis were also sorted out. The gene for acetyl-CoA *C*-acetyltransferase (EC: 2.3.1.9) and interestingly all mevalonate pathway genes were enriched in the endophytic bacterial community, namely for hydroxymethylglutaryl-CoA synthase (EC:2.3.3.10), mevalonate kinase (EC:2.7.1.36), hydroxymethylglutaryl-CoA reductase (EC:1.1.1.88), diphosphomevalonate decarboxylase (EC:4.1.1.33), phosphomevalonate kinase (EC:2.7.4.2), geranyl-diphosphate synthase (EC:2.5.1.1), isopentenyl-diphosphate delta-isomerase (EC:5.3.3.2), and farnesyl-diphosphate synthase (EC:2.5.1.10). Similarly, genes for squalene synthase (EC:2.5.1.21) and microsomal epoxide hydrolase (EC:3.3.2.9) were also enriched. The root tissue had a relatively higher abundance of these enzymes than other tissues in both male and female plants (Fig. 8).

## Discussion

*S. grosvenorii* is an economically important medicinal plant, primarily used as a natural sweetener, which is about 250 times sweeter than sugar (Itkin et al. 2016). Moreover, it is also the source of various natural products belonging to amino acids, flavonoids, terpenoids, and lignans having different therapeutic activities such as antioxidant, antitussive, antiasthmatic, expectorant, hypoglycemic, and hepatoprotective (Wu et al. 2022). Therefore, the endophytes associated with dioecious *S. grosvenorii* plants have special importance as a source of potential microbes for the improvement of plant growth and mogroside production and as a source of bioactive compounds. Further, the dioecious nature of *S. grosvenorii*, where the male and female plants are different, makes studying their associated endophytic communities more important. Here, efforts were made to understand the complete bacterial endophytic diversity of male and female *S. grosvenorii* plants. As the endophytes in other studies had plant-tissue specificity (Pandey et al. 2016a, 2022; Dastogeer et al. 2018; Fang et al. 2019; Li et al. 2020), therefore, different parts of male and female *S. grosvenorii* plants were considered in this study. For this, metagenomics was performed on the tissues collected from different parts of male and female plants, such as roots, stems, leaves, flowers, and fruits (pulp and seeds). It was noted that the roots of both the male and female plants exhibited the highest diversity. Several other studies have also documented greater diversity in root tissue compared to the phyllosphere of various plants, including examples such as tomato (Dong et al. 2019), *Arabidopsis* (Bodenhausen et al. 2013), and medicinal plants like *Bouvardia ternifolia* (Villalobos-Flores et al. 2021). Furthermore, the *Proteobacteria* phylum exhibited pronounced predominance in both male and female plants, while the *Acinetobacter* genus was detected across all tissues of both types of plants. The prevalence of *Proteobacteria* has been documented in numerous plant species, including examples such as *Pulsatilla chinensis* (Xing et al. 2023), *Rehmannia glutinosa* (Wu et al. 2018), *Cinnamomum camphora* (Zhang et al. 2020), *Oryza sativa* (Moronta-Barrios et al. 2017), and *Picrorhiza kurrooa* (Tamang et al. 2023), highlighting its widespread presence in various plants. The endophytic nature with plant growth-promoting properties of *Acinetobacter* has been well reported in many plants, including rice (Moronta-Barrios et al. 2017), *Papaver somniferum* (Pandey et al. 2016a), and sugar beet (Shi et al. 2011). Examining the unique populations in female and male plants, these bacterial communities have been extensively documented for their endophytic function, exhibiting numerous beneficial attributes for plants. Among distinct taxa associated with female plants, *Pseudomonas* and *Pantoea* were reported as core taxa enriched across many species such as *Salvia miltiorrhiza* (Chen et al. 2018), maize (Gao et al. 2019; Liu et al. 2020), bean (Klaedtke et al. 2016), and rice (Midha et al. 2016). Endophytic *Enterobacter* with antagonistic association towards plant fitness is well established (Witzel et al. 2012; Naveed et al. 2014; Andreozzi et al. 2019; Ullah et al. 2020). Bacteria belonging to *Methylotenera* had diverse metabolic capabilities (Kalyuzhnaya et al. 2010) and were also reported as endophytes in root samples of *Mentha longifolia* (Alreedy 2022). *Rheinheimera* has been reported as endophytes from the rhizosphere of *Hordeum secalinum* (Suarez et al. 2014), rice (Zhang et al. 2008), and the medicinal plant *Echinacea purpurea* (Presta et al. 2017). *Aeromonas* was the dominant genus in the roots of reed plants (Li et al. 2010), and it was also found to have the capability to reduce nitrate to nitrite (Minana-Galbis et al. 2007), imparting an important role in nitrogen cycling. *Macrococcus* endophytic diversity was reported in *Aloe vera* with antioxidant properties (Akinsanya et al. 2015) and pearl millet (Manjunatha et al. 2019). Seeds of Dongxiang wild rice are known to harbor *Facklamia* as an endophyte (Zhang et al. 2021) and also as a core microbe of the leaf endosphere of rice (Kumar et al. 2021), whereas *Propionibacterium* was found as a common endophyte in grapevines (Campisano et al. 2014).

Furthermore, in the case of distinct communities of male plants, endophytic *Herbaspirillum* was reported to colonize many plants such as rice (You et al. 2005; Andreozzi et al. 2019) and other members of the *Gramineae* family (Olivares et al. 1996) including tropical grasses (Pedrosa et al. 2011). *Duganella* is a well-reported endophyte from maize (Raths et al. 2021) and has been found to produce purple pigment violacein (Aranda et al. 2011). Endophytes belonging to the family *Hyphomicrobiaceae* with N-fixating properties were reported from the roots and rhizomes of *Miscanthus giganteus* (Liu and Ludewig 2019). Endophytes belonging to *Sphingomonadaceae* with plant-beneficial properties were reported from grapevine (Lòpez-Fernàndez et al. 2016), soybean (Okubo et al. 2009), *Juncus acutus* (Syranidou et al. 2018), *Caragana microphylla* (Dai et al. 2014), etc. *Lentzea* was one of the most abundant root endophytes in wheat plants (Elvia et al. 2021) and Chinese medicinal plants (Peng et al. 2015). *Mesorhizobium* imparted defense against pathogens and growth promotion to plants (Jabeen et al. 2016; Vijayabharathi et al. 2018; Nagpal et al. 2020). Endophytes belonging to *Rhodospirillaceae* were found to be a dominant community in the rhizosphere of sugarcane (de Souza et al. 2016). *Virgisporangium* formed an abundant part of the rhizosphere actinobacterial community in *Ainsliaea henryi*, a traditional medicinal plant (Zhao et al. 2012). *Paracoccus* was isolated from *Sphaerophysa salsula* with siderophore-producing properties (Deng et al. 2011), and a novel species of *Paracoccus* was also isolated from *Gastrodia elata* tubers (Zhang et al. 2019). *Legionellaceae* family endophytes in bananas were characteristic endophytes in healthy banana plants in comparison to *Fusarium* wilt-infected plants (Nakkeeran et al. 2021) and also in anthracnose asymptomatic leaves of *Pelargonium graveolens* (Da Silva et al. 2017), suggesting the role of endophytes in disease resistance. Similarly, the *Rhizobiales* order was also found as root-abundant endophytes in tomato (Tian et al. 2017) and *Miscanthus sinensis* (Sun et al. 2021). The presence of these endophytes with diverse plant-beneficial properties suggested the dynamic role of these communities in plant fitness and overall growth.

The core microbiome in this study was computed to understand the microbial composition, which was unchanged across both male and female plants. The understanding of the core microbiome has been gaining immense importance in the field of microbial ecology because it could be defined as microbial taxa-associated functional or genomic attributes that may be a unique characteristic of specific hosts or environments (Turnbaugh et al. 2007; Hamady and Knight 2009; Risely 2020). Interestingly, *Acinetobacter* was the most predominant in core taxa and also forms a part of core taxa in many other plants such as rice (Zhang et al. 2019; Kumar et al. 2021; Sahu et al. 2022) and *Eucommia ulmoides* (Dong et al. 2021). Along with that, the *Acinetobacter* genus has been extensively investigated due to its nitrogen-fixing capacity, synthesis of phytohormones, and ability to solubilize minerals, as evidenced by earlier studies (Rokhbakhsh-Zamin et al. 2011; Yuan et al. 2011). As evident as how the core microbiome is defined, this genus possibly plays a significant and stable role in plant development, which could be characteristic and essential for its holistic development. Linear discriminant analysis (LDA) was utilized to identify taxa that can be the potential biomarkers in different parts of the male and female *S. grosvenorii* plants. LEfSe first uses the Kruskal–Wallis test to identify the relatively abundant taxa in different groups, after which LDA that meets the significant threshold level is applied to estimate the effect size (Segata et al. 2011a). The genus representing different anatomical parts of both male and female plants as biomarkers could be instrumental in identifying the genders of the plant.

Predictive analysis of functional genes was conducted using PICRUSt. While this method offers a predictive insight into the gene composition within bacterial communities, the information generated in this study contributed to a novel comprehension and a holistic view of the distinct metabolic processes inherent in endophytic bacterial communities of *S. grosvenorii*. Notably, the gene for acetyl-CoA C-acetyltransferase was identified among the enriched genes. This enzyme facilitates the conversion of two acetyl-CoA to CoA and acetoacetyl-CoA, thereby serving as the pivotal entry point into the mevalonate pathway—the precursor pathway for mogrosides biosynthesis (Itkin et al. 2016). Similarly, the enrichment of terpenoid backbone genes such as for hydroxymethylglutaryl-CoA synthase, hydroxymethylglutaryl-CoA reductase, mevalonate kinase, phosphomevalonate kinase, diphosphomevalonate decarboxylase, isopentenyl-diphosphate delta-isomerase, geranyl-diphosphate synthase, and farnesyl-diphosphate synthase was also observed in the endophytic bacterial communities that indicated their role in mogroside biosynthesis in *S. grosvenorii* (Tang et al. 2011). Intriguingly, the gene encoding squalene synthase (catalyzes the formation of squalene, a fundamental precursor for triterpenoid and sterol biosynthesis) is present as a sole copy in the *Siraitia* genome (Itkin et al. 2016) and was also detected within the bacterial community. Therefore, the presence of the gene encoding squalene synthase in the endophytic bacterial community may help the host plant for the synthesis of mogroside. Similarly, the presence of the gene encoding cytochrome P-450 reductase (which is required by squalene epoxidase, epoxide hydrolase, and CYP87D18 involved in key steps of mogroside biosynthesis) in the associated bacterial endophytic community also indicated the involvement of endophytic bacterial community of *S. grosvenorii* in mogroside biosynthesis (Zhao et al. 2018). Another noteworthy gene enrichment pertaining to the identification of the gene encoding microsomal epoxide hydrolase, responsible for catalyzing the synthesis of trans-24,25-dihydroxycucurbitadienol—an indispensable intermediate in mogrosides biosynthesis, also indicated the association of endophytic community with the mogroside biosynthesis. Enrichment of genes involved in secondary metabolite biosynthesis such as the genes encoding glycogen synthase, glycogen phosphorylase, sucrose–phosphate synthase, trehalose 6-phosphate synthase, ADP-glucose-starch glucosyltransferase, 4-alpha-glucanotransferase, zeaxanthin glucosyltransferase, and alpha, alpha-trehalose phosphorylase in the associated endophytic communities indicated their role in plant secondary metabolite biosynthesis. The presence of genes encoding these enzymes in the associated endophytic bacterial community suggests an association between the endophytic microbes and the *S. grosvenorii* host plant in the biosynthesis of crucial secondary metabolites, especially mogroside biosynthesis. The presence of two endophytic strains, *Diaporthe angelica* LHG-F5, isolated from fruits, and *F. solani* LHG-L4, isolated from leaves of *S. grosvenorii* producing mogroside V, also indicates the role of associated endophytes in mogroside biosynthesis (Bin et al. 2020). These findings will help to understand the role of the microbial community in host metabolism and also pave the way to explore different microbial potentials in enhancing commercially important metabolites.

Hence, the possibility that endophytic bacterial secondary metabolism holds the potential to augment the production of secondary metabolites in *S. grosvenorii* may lead to a novel comprehension of the biosynthesis of essential metabolites of this medicinal plant. This phenomenon may be attributed to several factors: firstly, mogroside, a type of triterpenoid, and there is an investigation which suggests that both plants and endophytes share common metabolic processes and the biosynthesis pathway of terpenoids in endophytic bacteria bears similarity to that in plants, as indicated by studies such as those conducted by Goldstein and Brown (1990) and Bloch (1992). Secondly, the heightened metabolism of terpenoids and polyketides by endophytic bacteria may contribute directly to the increased accumulation of terpenoids in the plant by providing important precursor molecules. Moreover, as the terpenoids also play crucial roles in photosynthesis, plant growth, development, and intracellular signal transduction, therefore, the produced terpenoids collectively exert a synergistic influence on plant growth and secondary metabolism (Lange et al. 2000). Lastly, the pivotal link between endophytic bacteria and the biosynthesis of plant secondary metabolites lies in the intracellular signal transduction system. Endophytes also act as inducers that influence host metabolites during colonization. This interaction triggers the activation of signaling networks and other biological processes, ultimately affecting the expression of relevant genes and mediating the biosynthesis and accumulation of plant secondary metabolites. Noteworthy signaling molecules involved in these responses include jasmonic acid, salicylic acid, and hydrogen peroxide signaling, as documented by Wang et al. (2023).

Thus, the investigation represents a pioneering exploration of the endomicrobiome of monk fruit, specifically emphasizing the microbial diversity within the male and female plants. Investigating endophytes within the monk fruit plant is an underexplored domain, and our research addresses this gap by delving into the intricacies of endophytic diversity, a significant aspect in the realm of monk fruit research. The examination revealed the distinctive communities residing in various tissues of both male and female plants. Notably, unique microbial communities exclusive to the fruit, a commercially vital part of the plant with industrial applications and the primary site for mogroside accumulation, were also identified. Thus, the principal contribution of our investigation lies in its thorough exploration of endophytic diversity, providing valuable insights that enhance the overall understanding of the plant’s biology. Furthermore, our research offers essential preliminary findings, serving as a foundation for future studies that delve deeper into the implications for mogroside biosynthesis and the potential role of the host endomicrobiome. Functional gene prediction results indicated the presence of microbial communities with potential biosynthetic genes associated with mogroside production. Consequently, this study establishes a groundwork and lays the basis for more focused and detailed investigations in our subsequent research endeavors.

Therefore, in conclusion, the present study explored the understudied endomicrobiota of the dioecious *S. grosvenorii* monk fruit plant, where the male and female plants are separate and have distinct importance due to their involvement in monk fruit production as the female plants bear the fruits. Both male and female plants appear morphologically similar (except their flowers and fruit-bearing female plants) but have distinct bacterial endophytic communities associated with different parts of plants, and specific to male and female plants. Predictive functional analysis revealed the potential involvement of associated endophytic communities in the biosynthesis of host secondary metabolites. Therefore, male and female *S. grosvenorii* plant-associated endophytic microbial communities have promising potential to improve monk fruit cultivation, mogroside production, and early-stage identification of male and female plants. This study also intends to think about the consideration of both male and female plants of a dioecious plant for understanding/studying plant-microbial interactions.

## Supplementary Information

Below is the link to the electronic supplementary material.

Supplementary file1 (PDF 923 KB)

## Data Availability

The sequence files and corresponding metadata for all samples used in this study were deposited in the NCBI BioSample repository (Accession number PRJNA950226).
