# Supplementary material for: Unraveling endophytic diversity in dioecious Siraitia grosvenorii: implications for mogroside production
Source: Appl Microbiol Biotechnol. 2024 Mar 1;108:247. doi: 10.1007/s00253-024-13076-8 (PMC10907472; doi:10.1007/s00253-024-13076-8)
Supplement: Supplementary file 1 [file 253_2024_13076_MOESM1_ESM.pdf]

Applied Microbiology and Biotechnology

Supplementary Material

Unraveling endophytic diversity in dioecious *Siraitia grosvenorii*: implications for mogroside production

Anish Tamang[1,3,#], Amanpreet Kaur [1,#], Deepali Thakur [1,#], Ankita Thakur [1,3], Babit Kumar Thakur[2,3], Shivani[2,3], Mohit Swarnkar[1], Probir K. Pal[2,3], Vipin Hallan[1,3] and Shiv Shanker Pandey[1,3,*]

[1]Biotechnology Division, CSIR-Institute of Himalayan Bioresource Technology (IHBT), Palampur- 176061 (H.P), India.

[2]Division of Agrotechnology of Medicinal, Aromatic and Commercially Important Plants, Council of Scientific and Industrial Research-Institute of Himalayan Bioresource Technology (CSIR-IHBT), Post Box No. 6, Palampur, 176 061, HP, India

[3]Academy of Scientific and Innovative Research (AcSIR), Ghaziabad- 201002, India.

***Corresponding author: Shiv Shanker Pandey, E-mail: shivpandey@ihbt.res.in**

Table S1 Relative abundance of different taxa present in male and female *S. grosvenorii* plants

Taxonomic identification	% abundance	Taxonomic identification	% abundance
**Female plant**		**Male plant**	
**Root**		**Root**	
g__*Acinetobacter*	29.52	g__*Acinetobacter*	18.45
f_*Haliangiaceae*	10.24	f_*Haliangiaceae*	13.24
f_*Spirochaetaceae*	7.38	f_*Spirochaetaceae*	11.60
**g__*Pseudomonas***	8.45	f_*Enterobacteriaceae*	1.82
f_*Enterobacteriaceae*	5.48	c_*TM7-1*	9.37
**g_*Enterobacter***	3.93	**g_*Herbaspirillum***	8.44
f_*Bradyrhizobiaceae*	6.07	f_*Bradyrhizobiaceae*	4.92
g_*Ralstonia*	1.67	g_*Ralstonia*	1.52
**g_*Pantoea***	10.60	g_*Turneriella*	4.28
g_*Turneriella*	0.95	g_*Spirochaeta*	3.34
g_*Spirochaeta*	1.90	**g_*Duganella***	4.22
**p_*OD1***	9.64	**f_*Hyphomicrobiaceae***	3.93
p_*TM7*	3.33	**f_*Spingomonadaceae***	3.28
		**g_*Lentzea***	3.11
		**g_*Mesorhizobium***	2.64
		**f_*Rhodospirillaceae***	1.93
		**g_*Virgisporangium***	1.58
		**g_*Paracoccus***	0.76
		**f_*Legionellaceae***	0.59
		**g_*Turicibacter***	0.59
		**o_*Rhizobiales***	0.41
**Leaf**		**Leaf**	
g__*Acinetobacter*	1.80	g__*Acinetobacter*	83.18
**g__*Pseudomonas***	48.65	**g_*Vogesella***	16.82

f_*Enterobacteriaceae*	15.02		
**g_*Enterobacter***	34.54		
**Stem**		**Stem**	
g__*Acinetobacter*	32.88	g__*Acinetobacter*	23.02
**f_*Methylobacteriaceae***	7.53	**g__*Bacillus***	49.21
**g_*Methylotenera***	23.97	**g_*Arthrobacter***	27.78
**g_*Rheinheimera***	21.92		
**g_*Aeromonas***	13.70		
**Flower**		**Flower**	
g__*Acinetobacter*	17.74	g__*Acinetobacter*	100.00
f__*Enterobacteriaceae*	82.26		
**Fruit pulp**			
g__*Acinetobacter*	33.72		
**g__*Macrococcus***	47.26		
g_*Ralstonia*	10.09		
**g_*Facklamia***	5.76		
**g__*Propionibacterium***	3.17		
**Fruit seeds**			
f__*Enterobacteriaceae*	31.21		
g__*Ralstonia*	17.34		
**p__*Proteobacteria***	32.95		
**f__*Methylobacteriaceae***	18.50		

Distinct groups specific to male and female plants are written in bold

Table S2 LEfSe analysis statistical data for each taxon by sample groups as calculated for presentation in Fig. 5

	p values	FDR	Female flower	Female fruit	Female leaf	Female root	Female stem	Male flower	Male leaf	Male root	Male stem	Seed female plant	LDA score
Pseudarthrobacter	0.000672	0.001756	0	0	0	0	0	0	0	0	442750	0	5.35
Bacillus	0.000672	0.001756	0	0	0	0	0	0	0	0	3696200	0	6.27
Pseudomonas	0.000675	0.001756	0	0	6667800	776970	0	0	0	0	0	0	6.52
Escherichia_Shigella	0.001744	0.003779	0	0	1059900	51282	0	0	0	79794	0	1156000	5.76
Acinetobacter	0.002155	0.003978	8321400	9101400	274780	7732300	9313100	8862800	1.00E+07	7853800	3005600	3737100	6.69
Methylobacterium	0.002753	0.003978	0	0	0	0	387710	0	0	0	0	1884600	5.97
Ralstonia	0.003058	0.003978	1678600	898640	0	221410	299230	998290	0	368470	129630	2942600	6.17
Enterobacter	0.003292	0.003978	0	0	1997500	343380	0	0	0	65493	0	279610	6
Rhodomicrobium	0.003661	0.003978	0	0	0	17094	0	0	0	485670	0	0	5.39
Pantoea_agglomerans	0.000672	0.003497	0	0	0	1739100	0	0	0	0	0	0	5.94
Massilia	0.000672	0.001266	0	0	0	0	0	0	0	288870	0	0	5.16

(FDR: false discovery rate)

Table S3 Different pathways are enriched through functional prediction of genes from the endophytic bacterial community.

Pathway	Size	Hits
Phenylpropanoid biosynthesis	2	2
Novobiocin biosynthesis	4	4
Tropane, piperidine and pyridine alkaloid biosynthesis	5	5
Fructose and mannose metabolism	33	33
Ascorbate and aldarate metabolism	17	17
Teichoic acid biosynthesis	3	3
Naphthalene degradation	5	5
Chloroalkane and chloroalkene degradation	7	7
Retinol metabolism	2	2
Glycosaminoglycan degradation	2	2
Other glycan degradation	2	2
Lipopolysaccharide biosynthesis	19	19
Ubiquinone and other terpenoid-quinone biosynthesis	22	22
Isoquinoline alkaloid biosynthesis	5	5
Phosphonate and phosphinate metabolism	6	6
Biosynthesis of unsaturated fatty acids	2	2
Lysine degradation	16	16
Polycyclic aromatic hydrocarbon degradation	3	3
Vitamin B6 metabolism	9	9
Sphingolipid metabolism	5	5
Prodigiosin biosynthesis	3	3
Benzoate degradation	25	25
Glycolysis / Gluconeogenesis	26	26
Citrate cycle (TCA cycle)	12	12
Arginine biosynthesis	19	19
Terpenoid backbone biosynthesis	17	17
Tyrosine metabolism	27	27
Biotin metabolism	13	13

Pyruvate metabolism	33	33
Fatty acid biosynthesis	11	11
Tryptophan metabolism	19	19
Arginine and proline metabolism	42	42
Glycerolipid metabolism	14	14
Carbon fixation in photosynthetic organisms	17	17
Fatty acid degradation	14	14
Butanoate metabolism	32	32
Galactose metabolism	22	22
Carbapenem biosynthesis	2	2
Carotenoid biosynthesis	7	7
Alanine, aspartate and glutamate metabolism	23	23
Starch and sucrose metabolism	32	32
Linoleic acid metabolism	2	2
Phenylalanine metabolism	27	27
Nitrogen metabolism	13	13
Pantothenate and CoA biosynthesis	18	18
Pentose phosphate pathway	30	30
Phenylalanine, tyrosine and tryptophan biosynthesis	20	20
Dioxin degradation	4	4
Nicotinate and nicotinamide metabolism	16	16
Carbon fixation pathways in prokaryotes	22	22
Glycerophospholipid metabolism	15	15
Valine, leucine and isoleucine degradation	25	25
Limonene and pinene degradation	3	3
Purine metabolism	60	60
Styrene degradation	11	11
Sulfur metabolism	14	14
Glyoxylate and dicarboxylate metabolism	41	41
beta-Alanine metabolism	19	19

Phenazine biosynthesis	2	2
Amino sugar and nucleotide sugar metabolism	50	50
Lipoic acid metabolism	3	3
Atrazine degradation	3	3
Pentose and glucuronate interconversions	28	28
Histidine metabolism	16	16
Glucosinolate biosynthesis	2	2
Taurine and hypotaurine metabolism	6	6
Thiamine metabolism	18	18
Biosynthesis of vancomycin group antibiotics	2	2
Cysteine and methionine metabolism	44	44
Acarbose and validamycin biosynthesis	2	2
Ethylbenzene degradation	2	2
O-Antigen nucleotide sugar biosynthesis	23	23
Folate biosynthesis	23	23
Aminobenzoate degradation	8	8
Lysine biosynthesis	15	15
Cyanoamino acid metabolism	6	6
Propanoate metabolism	33	33
Xylene degradation	7	7
Valine, leucine and isoleucine biosynthesis	9	9
Oxidative phosphorylation	9	9
Metabolism of xenobiotics by cytochrome P450	3	3
Biosynthesis of various plant secondary metabolites	3	3
alpha-Linolenic acid metabolism	3	3
Drug metabolism - cytochrome P450	4	4
D-Amino acid metabolism	15	15
Inositol phosphate metabolism	15	15
Pyrimidine metabolism	39	39
Glutathione metabolism	14	14

Drug metabolism - other enzymes	11	11
Geraniol degradation	4	4
Streptomycin biosynthesis	9	9
Glycine, serine and threonine metabolism	37	37
Methane metabolism	32	32
Ether lipid metabolism	3	3
Selenocompound metabolism	9	9
Polyketide sugar unit biosynthesis	4	4
Glycosphingolipid biosynthesis - globo and isoglobo series	2	2
Porphyrin metabolism	42	42
Monobactam biosynthesis	7	7
Toluene degradation	4	4
Caprolactam degradation	5	5
One carbon pool by folate	11	11
C5-Branched dibasic acid metabolism	7	7
Riboflavin metabolism	14	14
Chlorocyclohexane and chlorobenzene degradation	6	6
Peptidoglycan biosynthesis	12	12
Fluorobenzoate degradation	6	6

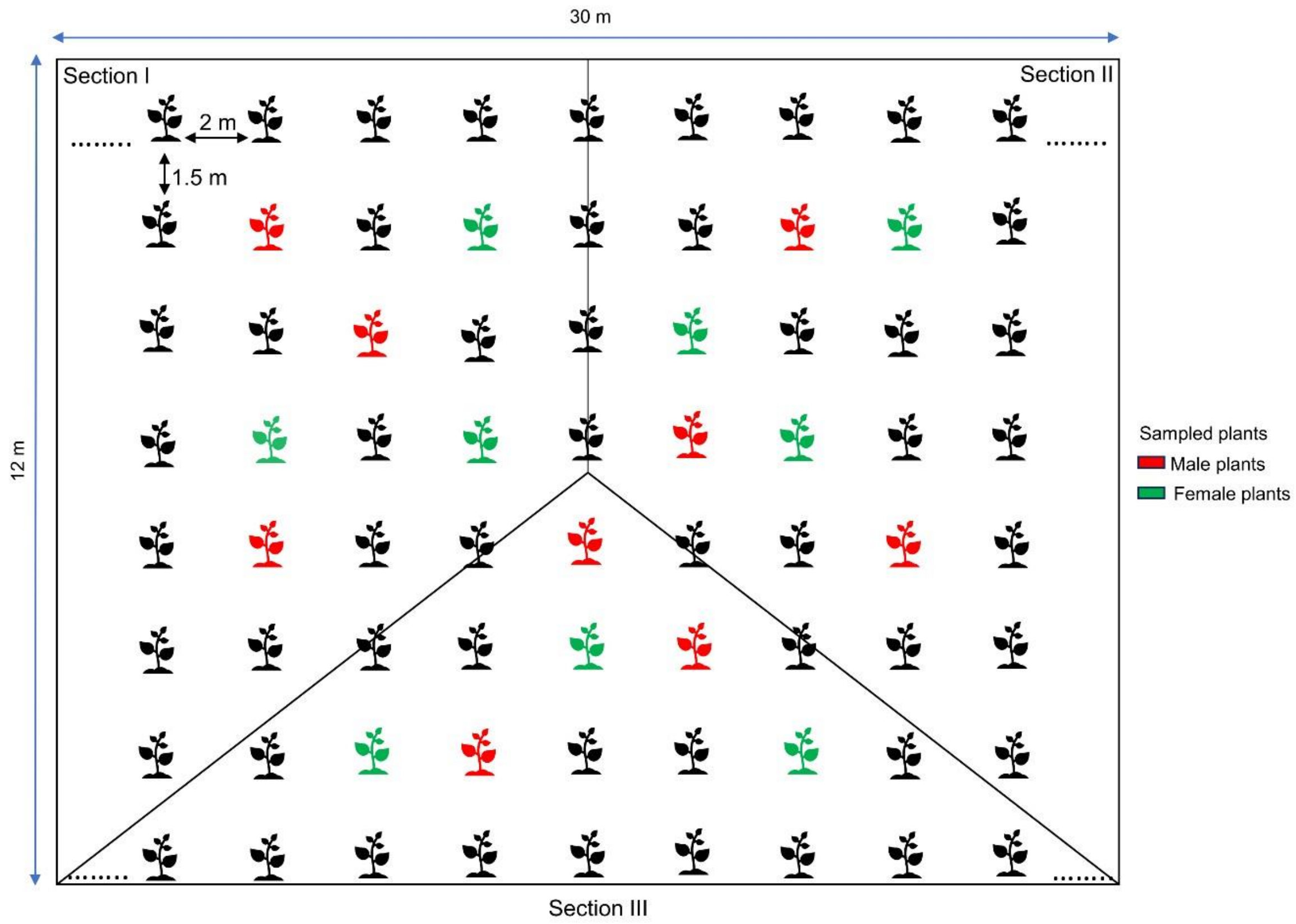

Fig. S1 Sampling layout for collection of samples for sequencing. Plot depicting *Siraitia grosvenorii* plants cultivated in the farm at CSIR-IHBT, Palampur, where both male and female plants were grown randomly. For sampling, the plot was divided into three sections and from each section, 3 male plants (red coloured) and 3 female plants (green coloured) were taken for sampling. Therefore, a total of 9 male plants and 9 female plants were taken for harvesting their samples of respective plant parts. Samples (each plant-part of a plant) of 3 plants (collected from each section) were pooled and considered as one replicate and a total of 3 replicates were used for sequencing for each plant-part of both male and female plants.

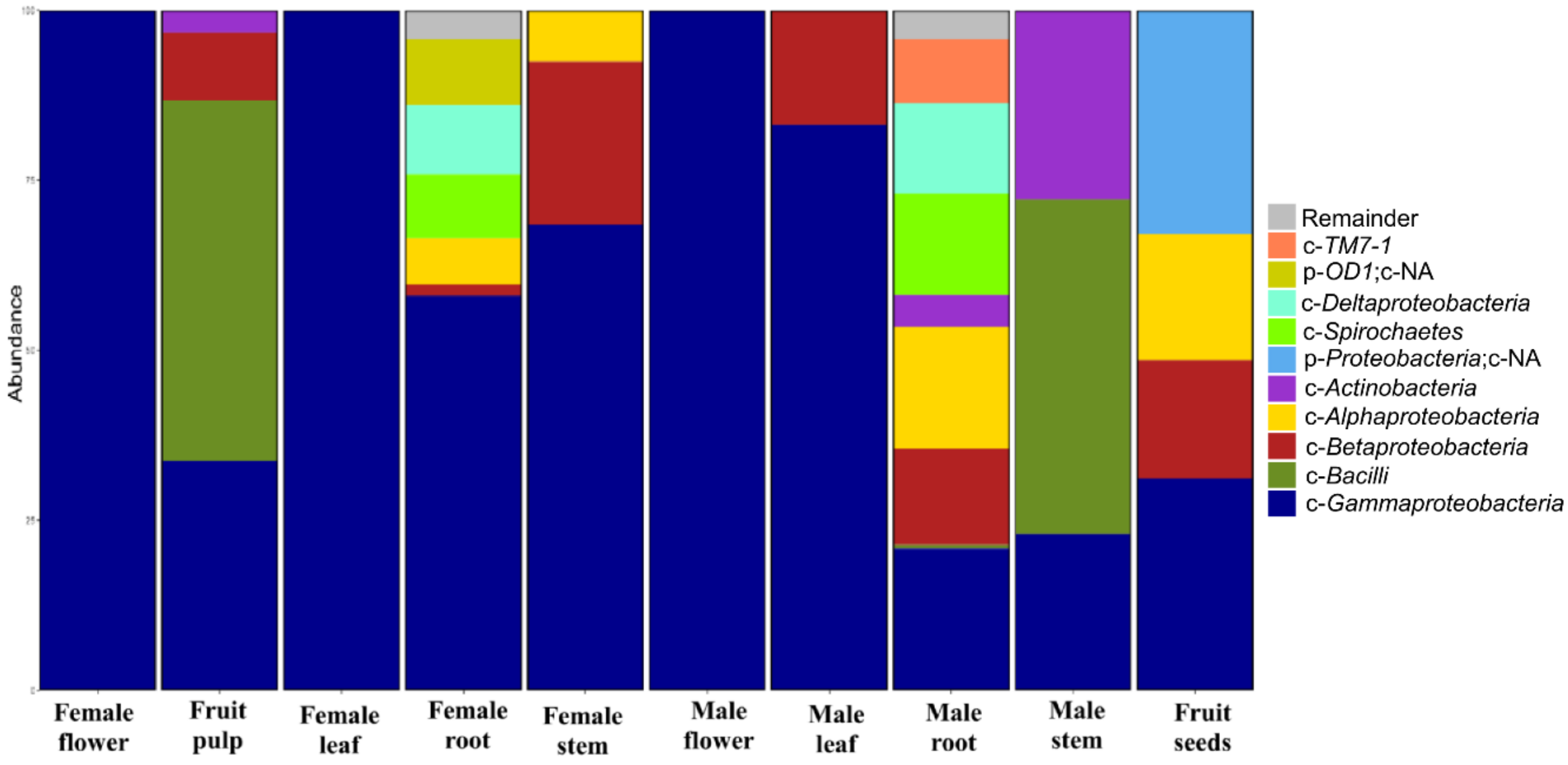

Fig. S2 Relative abundance of different taxa at class level in different parts of male and female *S. grosvenorii* plants.

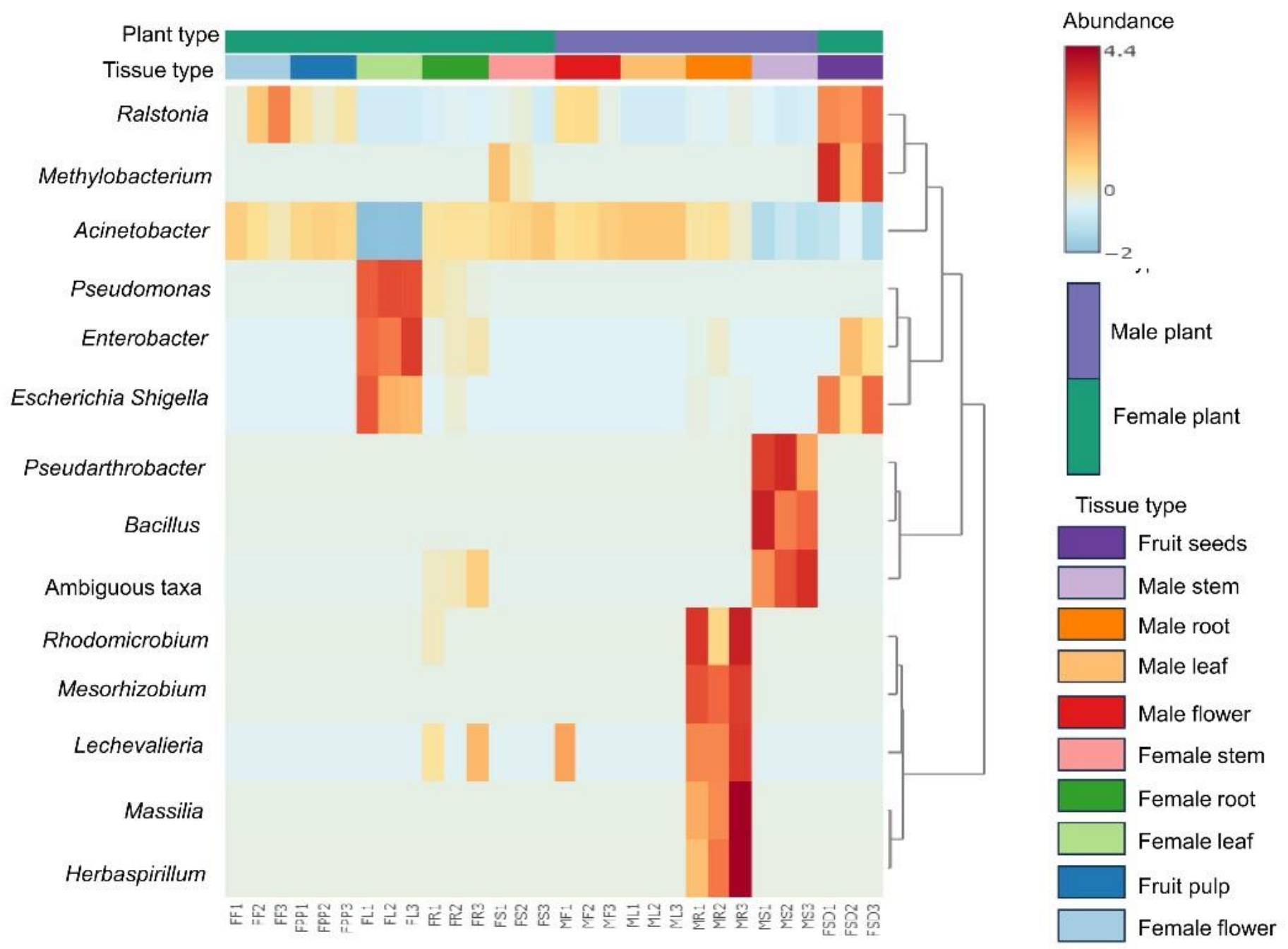

Fig. S3 Heat map depicts the abundance of different taxa in various tissues of male and female plants of *S. grosvenorii.* (FF1-FF2: Female flower; FPP1-FPP3: Fruit pulp; FL1-FL3: Female leaf; FR1-FR3: Female root; FS1-FS3: Female stem; MF1-MF3: Male flower; ML1-ML3: Male leaf; MR1-MR3: Male root; MS1-MS3: Male stem; FSD1-FSD3: Fruit seeds)
